# Editorial: Pathological hyperactivity and hyperexcitability in the central nervous system

**DOI:** 10.3389/fnmol.2022.955542

**Published:** 2022-07-12

**Authors:** Michael Telias, Menahem Segal

**Affiliations:** ^1^Flaum Eye Institute, University of Rochester, Rochester, NY, United States; ^2^Department of Neurobiology, The Weizmann Institute of Science, Rehovot, Israel

**Keywords:** hyperexcitability, hyperactivity, fragile X syndrome, Alzheimer's disease, neuron, brain, epilepsy

The brain contains a delicate and precise balance between excitatory and inhibitory neurons, which have different roles in the formation, development, and homeostasis of brain circuits. The balance between these populations is maintained by a number of local control mechanisms, regulating presynaptic efficacy as well as postsynaptic excitability. In pathology, an abnormal excitation-to-inhibition (E/I) ratio leads to neuronal hyperexcitability and network hyperactivity, constituting the basis for epilepsy and subsequent cognitive dysfunction, and is associated with many neurodevelopmental, neurodegenerative, and psychiatric diseases. Epileptic seizures can be induced by blocking GABAergic inhibition and repressed by enhancing inhibition, illustrating the role of E/I-ratio imbalance in neuronal pathology (Smolarz et al., 2021; Vlachou, 2022). In this Topic, the contributing studies showcase the centrality of hyperexcitability and hyperactivity in five different pathologies with diverse etiologies and symptoms.

## Fragile X syndrome

In recent years, much attention has been focused on neurodevelopmental disorders including autism spectrum disorders, Rett syndrome, and Angelman syndrome which are accompanied by epileptic seizures, sensory hypersensitivity, repetitive behavior, and attention deficits; each symptom potentially caused by independent mechanisms (Antoine). These diseases all share phenotypic traits with the archetype of developmental disorders, fragile X syndrome (FXS) (Telias, 2019), a monogenic X-linked disorder. For example, neuronal hyperexcitability can explain sensory hypersensitivity in FXS patients. Here, Deng et al. propose that reduced expression and activation of HCN channels contributes to hyperexcitability of dorsal root ganglia in the *fmr1*^−/−^ mouse; while the study by Avraham et al. shows a role in FXS-hypersensitivity for altered neuron-glia interactions, in which *fmr1*^−/−^ satellite glial cells fail to properly contact and cover the dorsal root ganglia. Beyond sensory processing, other cognitive functions are affected by hyperactivity in FXS. The in-depth review by Liu et al. provides an excellent analysis of the latest research on the subject, with an emphasis on molecular and physiological mechanisms, overall suggesting that excitability and homeostatic plasticity are both impaired in FXS, in a region-selective manner.

## Hearing loss

In hearing loss, as the hair cells in the cochlea die, the auditory nerve becomes hyperactive, giving rise to tinnitus (Shore et al., 2016). This increase in firing has repercussions in the downstream brain nuclei that receive cochlear inputs, and understanding them is essential to improve hearing restoration strategies. Downstream to the cochlea, circuits cross into the contralateral hemisphere, forming the main auditory pathway. Interneurons in the brain stem carry signals from the terminals of spiral ganglion cells to the contralateral superior olive nucleus, and from there the fibers ascend to the inferior colliculus. Yet, evidence for the existence of ipsilateral pathways and their roles is emerging. Hsiao and Galazyuk demonstrate that the pathological hyperactivity that results from unilateral sound exposure affects both the ipsi- and the contralateral pathways, but the largest effect was measured in the ipsilateral inferior colliculus, an unexpected result that challenges existing paradigms, as most studies using unilateral acoustic damage focus almost exclusively on the nuclei of the contralateral side. Further downstream effects of cochlear damage and subsequent hyperactivity can affect sensory gating in the auditory system. De Vis et al. show that acoustic damage to the cochlea in guinea pigs affects signal transmission between the pre-frontal cortex and the medial geniculate nucleus, essential for gating of sensory input, providing a physical substrate for tinnitus. Since the prefrontal cortex is involved in sensory gating for many different pathways other than audition, their results could have far-reaching consequences.

## Alzheimer's disease

Neuronal hyperexcitability and network hyperactivity are intrinsically related to Alzheimer's Disease (AD) progression, in both sporadic and familial AD (Palop and Mucke, 2016; Kazim et al., 2021). Hypersynchrony in the brains of AD patients can be observed long before clinical symptom manifestations, increasing the risk for epilepsy and faster cognitive decline, while hyperexcitability has been linked to activation and accumulation of β-amyloid and tau. The source of the hyperactivity in AD remains unknown, as animal models show defects in both inhibition and excitation. In an age-dependent rat model of AD, enhanced excitability is partially caused by increased activity of β-adrenergic receptors (Goodman et al., 2021). Now, Smith et al. show that hippocampal neurons in AD become hyperexcitable due to intrinsic changes in membrane properties, independent of changes in pre-synaptic input. The study shows that affected neurons have increased input resistance, deceased rheobase, and lower firing thresholds, together with a decrease in sag associated with functional upregulation of HCN channels, providing new insights into the mechanisms of hyperexcitability in AD.

## Hyperexcitability and hyperactivity in pain and psychomotor behavior

The study by Kearns et al. demonstrates that hypersensitivity to pain (hyperalgesia), induced by chronic exposure to opioids, is mediated by specific alternations in excitatory and inhibitory spinal neurons. Chronic treatment with morphine was associated with both enhanced excitation of excitatory neurons, as well as with reduced excitation of inhibitory neurons. The effect seems to be mediated by changes in neurotransmitter-dependent mechanisms, as treatment with morphine did not alter most passive membrane properties in any of the tested cell populations. The study by Zhang et al. discusses hyperactivity in the context of locomotion and psychomotor behavior. Inhibition of 14-3-3 protein in the hippocampus induces locomotor hyperactivity that is mitigated by pharmacological blockade of dopamine and by inhibition of firing of dopamine neurons in the ventral tegmental area. This work also identifies the lateral septum as a relay station between the ventral tegmental area and the hippocampus.

## Afterword

This Frontiers Topic highlights the versatility of hyperactivity and hyperexcitability in neurological disease, observed in neurodevelopmental and neurodegenerative disorders; as a consequence of intrinsic changes in neuronal properties or due to circuit-wide changes affecting excitation and/or inhibition. The mechanisms underlying each case might differ, but the phenotypic outcome remains the same. Most importantly, for each individual case, it is still unclear whether hyperactivity is just a consequence of an imbalanced system, or is in itself the source of further dysfunctionality by amplifying the defects associated with the disease.

## Author contributions

MT and MS contributed equally to the writing and editing of this manuscript. Both authors contributed to the article and approved the submitted version.

## Funding

MT was supported by an unrestricted gift from the Research for Preventing Blindness Foundation to the Flaum Eye Institute.

## Conflict of interest

The authors declare that the research was conducted in the absence of any commercial or financial relationships that could be construed as a potential conflict of interest.

## Publisher's note

All claims expressed in this article are solely those of the authors and do not necessarily represent those of their affiliated organizations, or those of the publisher, the editors and the reviewers. Any product that may be evaluated in this article, or claim that may be made by its manufacturer, is not guaranteed or endorsed by the publisher.
